# Neurotrophin-conjugated nanoparticles prevent retina damage induced by oxidative stress

**DOI:** 10.1007/s00018-017-2691-x

**Published:** 2017-11-02

**Authors:** Martina Giannaccini, Alice Usai, Federica Chiellini, Viviana Guadagni, Massimiliano Andreazzoli, Michela Ori, Massimo Pasqualetti, Luciana Dente, Vittoria Raffa

**Affiliations:** 1 Department of Biology, Università di Pisa, 56127 Pisa, Italy; 2 Department of Chemistry and Industrial Chemistry, Università di Pisa, 56124 Pisa, Italy

**Keywords:** Neurotrophins, Ocular drug delivery, Magnetic nanoparticles, Zebrafish, Glaucoma

## Abstract

Glaucoma and other optic neuropathies are characterized by a loss of retinal ganglion cells (RGCs), a cell layer located in the posterior eye segment. Several preclinical studies demonstrate that neurotrophins (NTs) prevent RGC loss. However, NTs are rarely investigated in the clinic due to various issues, such as difficulties in reaching the retina, the very short half-life of NTs, and the need for multiple injections. We demonstrate that NTs can be conjugated to magnetic nanoparticles (MNPs), which act as smart drug carriers. This combines the advantages of the self-localization of the drug in the retina and drug protection from fast degradation. We tested the nerve growth factor and brain-derived neurotrophic factor by comparing the neuroprotection of free versus conjugated proteins in a model of RGC loss induced by oxidative stress. Histological data demonstrated that the conjugated proteins totally prevented RGC loss, in sharp contrast to the equivalent dose of free proteins, which had no effect. The overall data suggest that the nanoscale MNP-protein hybrid is an excellent tool in implementing ocular drug delivery strategies for neuroprotection and therapy.

**Electronic supplementary material:**

The online version of this article (10.1007/s00018-017-2691-x) contains supplementary material, which is available to authorized users.

## Introduction

Neurotrophins (NTs) are known to promote neuronal survival and regeneration in both the central (CNS) and peripheral nervous system (PNS) [1]. The exogenous delivery of NTs has also been proposed for the treatment of optic neuropathies [2]. Optic neuropathies are a vast group of diseases. Glaucoma is the most common, and is characterized by visual loss due to optic nerve dysfunction.

The loss of retinal ganglion cells (RGCs) is a common feature of neuropathies, irrespectively of the aetiology of the disease. Retinal ganglion cells are a type of neuron located near the inner surface of the retina, in the ganglion cell layer (GCL), which integrates visual information from photoreceptors (Ph) and projects it to the brain. The nerve growth factor (NGF) and brain-derived neurotrophic factor (BDNF) are members of the NT family, which have been shown to prevent damage to GCL. NGF promotes the survival and recovery of retinal ganglion cells [3, 4]. It has been demonstrated that the injection of exogenous NGF protects retinal ganglion cells from degeneration and apoptosis in different experimental models of retinal detachment, diabetic retinopathy and glaucoma [5–7]. Clinical studies suggest that the administration of NGF eye drops improves all parameters of visual function in patients with advanced glaucoma [8]. Similarly, BDNF enhances the survival of GCL in vitro and in vivo [9–11]. BDNF also prevents the in vivo cell death of GCL induced by optic nerve lesions, providing therapeutic neuroprotection [12, 13].

The key limitation of NTs in the treatment of PNS and CNS diseases is their inability to cross the blood–brain barrier. Similarly, the eye has its own barriers. It is composed of two segments enveloped by the cornea and sclera: the anterior segment, which includes the cornea, the aqueous humour and the iris, and the posterior segment, which contains the lens, the vitreous humour, the neural retina (NR), the retinal pigment epithelium (RPE), and the choroid. The poor permeability of cornea/sclera and the blood retinal barrier make the systemic administration of NTs ineffective in reaching the retina.

A variety of approaches associated with the delivery of NTs to the retina have been proposed for local administration, including direct delivery, viral gene therapy, and cell-based therapy, which improve the development, survival, and function of retinal ganglion cells [14]. Renexus^®^ (NT-501) is an intravitreal (IVT) implant encapsulating the human RPE cell line genetically modified to secrete the ciliary neurotrophic factor (CNTF). It is currently in phase 2 of a clinical trial for the treatment of glaucoma (NCT02862938), early-stage retinitis pigmentosa (NCT01530659), and macular telangiectasia (NCT03071965). However, the direct delivery of proteins through IVT injections is the safest, most effective and common approach tested to date. Repeated IVT injection of anti-vascular endothelial growth factor agents (bevacizumab, ranibizumab, or pegaptanib) has become a gold standard anti-angiogenic treatment in many clinics [15].

However, the real potential of direct IVT injection of NTs as neuroprotective/restorative drugs in ophthalmology, has yet to be proven and many limitations still prevent its application in clinics. NTs have a very short half-life in vivo and unfavourable kinetics, with a very high initial peak of drug concentration at the time of administration, rapidly followed by a decline over time (minutes). Multiple IVT injections are thus required to obtain therapeutic efficacy, eliciting a concrete risk of cataracts, retinal ischemia and endophthalmitis [16]. Another serious problem related to multiple injections has been described by Chen and Weber, who showed that multiple applications of BDNF do not have an additive effect because the continuous application of BDNF intravitreally leads to the downregulation of its receptor [17].

To overcome problems of a short half-life, dosing and the undesirable effects of multiple injections, the incorporation of BDNF in a hyaluronic acid aqueous solution has been proposed [3]. The use of IVT injected nanoformulations has also been explored, such as the injection of basic fibroblast growth factor (FGF)-impregnated nanoparticles (NP) [4] or poly-lactic-*co*-glycolic acid (PLGA) microspheres containing NTs [18]. A single IVT injection of the glial cell-derived neurotrophic factor (GDNF) encapsulated in vitamin E/PLGA microspheres was found to protect RGCs in animal models of glaucoma for up to 11 weeks by providing the sustained controlled release of GDNF in a controlled fashion for up to 6 months [19, 20].

In previous studies, we demonstrated that magnetic nanoparticles (MNPs) could be used to bypass the limitations related to the IVT injection of free proteins [21, 22]. MNPs are largely used in biomedicine. They are biodegradable nanoparticles, which typically consist of a superparamagnetic central core of iron oxide surrounded by an organic coat. Several generations of MNPs have already been approved by the FDA for use on humans as contrast agents for magnetic resonance imaging or for the treatment of chronic anaemia [23].

We found that IVT-injected MNPs autonomously localise themselves in the retina where they persist for at least 3 weeks [21, 22]. In principle, MNP-mediated drug delivery could provide a therapeutically effective concentration of the carried molecule for weeks in the right location, thereby preventing side effects in other tissues. In addition, the molecule localization mediated by MNPs could prevent mass losses due to molecule degradation or physiological elimination by the humour vitreous.

In this paper, we demonstrate that NGF and BDNF conjugation to MNPs increases their stability. The functionalized particles are localized in the retina. We tested the neuroprotection of conjugated NTs, demonstrating that their IVT administration totally prevents retinal ganglion cell loss induced by oxidative stress. Interestingly, we used NGF and BDNF concentrations that were tenfold lower than the effective dose proposed in the literature and, in agreement with the previous data, we found that free NTs had no neuroprotective effects, in sharp contrast to the conjugated NTs.

## Materials and methods

### Functionalization of MNPs

Commercial MNPs were used (FluidMAG-ARA 4115, Chemicell). According to the datasheet, MNPs have a magnetite core of iron oxide (50 nm in size) and an organic shell which exposes –COOH groups. The particles were functionalized with NGF 2.5S (Alomone, N100) or fluorescently labelled NGF (NGF^fluo^) or recombinant BDNF or bovine serum albumin (BSA, Sigma, A2153). NGF^fluo^ was produced as already described [24], by labelling 100 µg of NGF 2.5S with Alexa Fluor 488 (Life Science, A10235), according to the manufacturer’s instructions. Purification through a size exclusion resin enabled the uncoupled dye to be discarded. The degree of labelling was about 1.5 mol of dye per mole of protein, and the efficiency of the labelling process was about 90% (*n* = 3).

The functionalization of the particles was carried out using an MNP:protein ratio of 3.5:1 w/w. For NGF and NGF^fluo^, we adopted a non-covalent approach as previously described [24–26]. Briefly, the MNP:protein suspension was incubated at 4 °C for 2 h with stirring. For BDNF and BSA, particles were covalently functionalized via EDC chemistry, according to a protocol that we optimized in previous studies with other proteins [22, 25, 26]. Briefly, particles were centrifuged (18,000*g*) and resuspended in 4% EDC (Sigma) water solution. After 10 min, the protein was added and mixed for 1 h at 4–8 °C. In both protocols, the unbounded protein was removed by centrifugation (18,000*g*) and by discharging the supernatant (two washing steps).

The amount of protein bound to the surface of MNPs was calculated by subtraction, i.e., by measuring the protein concentration (Bradford assay) of the supernatant derived from the washing steps. The amount of magnetic nanoparticles was quantified using the thiocyanate assay, according to a protocol we published previously [27]. Briefly, 3 µl of particle suspension was resuspended in 50 μl of a solution of 6 M HCl: 65% v/v HNO_3_ and incubated at 60 °C for 1 h. The sample was water diluted 1:10, an equal volume of 1.5 M KSCN was added, and absorbance recorded at 478 nm. The calibration curve was obtained with a known amount of MNPs (*y* = 0.055*x*, *R* ^2^ = 1), where y is the absorbance at 478 nm and x is the MNP concentration (mg/ml). The conjugated nanoparticles and the free proteins were stored in 20% glycerol at − 20 °C. The composition of MNP–NGF was 9.50 ± 0.11 mg/ml of MNPs, 0.90 ± 0.01 mg/ml of NGF, 20% glycerol (*n* = 4). The composition of MNP–NGF^fluo^ was 14 ± 0.15 mg/ml of MNPs, 0.43 ± 0.19 mg/ml of NGF, 20% glycerol (*n* = 4). The composition of MNP–BDNF was 13.88 ± 0.24 mg/ml of MNPs, 1.03 ± 0.15 mg/ml of BDNF, 20% glycerol (*n* = 4). The composition of MNP-BSA was 11.76 ± 0.14 mg/ml of MNPs, 0.90 ± 0.13 mg/ml of BSA, 20% of glycerol (*n* = 4).

The functionalized particles were characterized in terms of hydrodynamic size and Z potential by Delsa™NanoC (Beckman Coulter Fullerton, CA, USA).

### Cell cultures

Rat pheochromocytoma PC12 cells obtained from the American Type Culture Collection (ATCC) were cultured in Dulbecco’s modified Eagle’s medium (DMEM) with 10% horse serum, 5% foetal bovine serum (FBS), 100 IU/ml penicillin, 100 µg/ml streptomycin, and 2 mM l-glutamine. Cells were cultured in plastic coated with poly-l-lysine (PLL, Sigma, P1274) and maintained at 37 °C in a saturated humidity atmosphere of 95% air and 5% CO_2_. For cell differentiation, PC12 cells were incubated for 4 days in serum-reduced media (1% FBS), modified with MNP–NGF or free NGF (100 ng/ml). For Western blot (WB) analysis, PC12 cells were starved for 5 h in Optimem (Gibco) and incubated for 5 min in serum-reduced media unmodified (control) or modified with MNP–NGF or NGF (100 ng/ml). WB analysis was performed on protein extracts from PC12 cells using phospho-TrkA (Tyr490) antibody (Cell Signaling, 9141) and β-actin antibody (Sigma, A1978).

The human neuroblastoma SH-SY5Y cell line obtained from ATCC was cultured in DMEM/F12 medium with 10% FBS, 100 IU/ml penicillin, 100 µg/ml streptomycin and 2 mM l-glutamine. For cell differentiation, SH-SY5Y cells were incubated for 5 days in a cell growth medium modified with 10 µM retinoic acid (RA). Neurite networking was then induced by incubating cells for 5 days in DMEM unmodified (control) or modified with MNP–BDNF or free BDNF (100 ng/ml). Neurite networking was calculated by counting the number of interconnected neurites (i.e., neurites connected with at least one other neurite) over the total number of neurites (*n* = 5, 250–300 neurite per replicate).

### Stability in medium

Free or conjugated NGF was added to protease-rich medium (70% FBS, not inactivated) at a final concentration of 10 µg/ml. The medium was incubated at 37 °C for 0, 4, 10 or 14 days. Aliquots were collected at different time points and used to prepare the differentiation medium for inducing PC12 cell differentiation.

Free or conjugated BDNF was added to DMEM at a final concentration of 100 ng/ml. The medium was incubated at 4 °C for 0, 3 or 6 days. Aliquots from the different time points were used to induce network formation in RA-differentiated SH-SY5Y cells.

After incubation, cells were fixed in 2% paraformaldehyde for 20 min and stained with 0.05% crystal violet.

### Embryo preparation

Animal procedures were performed in strict compliance with protocols approved by the Italian Ministry of Public Health and of the local Ethical Committee of the University of Pisa (authorization 99/2012-A, 19.04.2012), in compliance with EU legislation (Directive 2010/63/EU). Zebrafish embryos (roy−/−; nacre−/− and AB) were obtained by natural mating and raised according to the ZFIN procedures (authorization 1173/2015-PR). Before any injection, larvae were anesthetized in 0.02% tricaine and embedded in 0.3% agarose.

### Localization studies

Two nl containing 10 or 20 ng of MNPs or MNP–NGF or MNP–BDNF were microinjected into the left eye of a four-day post-fertilization (dpf) larvae and fixed at 24 h post injection (hpi). Zebrafish larvae were fixed in 4% paraformaldehyde for 1 h, embedded in paraffin and sectioned (5 µm). The paraffin sections were stained by Prussian blue according to the manufacturer’s instructions (Sigma-Aldrich, St. Louis, USA) after a treatment of pigment bleaching in 5% formamide-1% hydrogen peroxide in the presence of cold light. The number of events (presence of staining in NR and/or RPE and/or choroid, Fig. S1) was counted and data were plotted after normalization.

In another experiment, 2 nl containing 10 ng of MNP–NGF^fluo^ were microinjected into the left eye of 4 dpf larvae and fixed at 6 hpi. Sections were imaged in bright field and in FITC channel.

Each experiment was performed on at least 15 larvae per group.

### Oxidative stress model

Two nl of H_2_O_2_ (0, 0.25, 0.5 or 1 M) were injected into the left eye of 5 dpf larvae. After 2 or 4 or 8 or 24 hpi, larvae were fixed in 4% paraformaldehyde for 1 h, embedded in paraffin, and sectioned (5 µm). The paraffin sections were stained with the DeadEnd™ Fluorometric TUNEL System (Promega Corporation, USA) and Hoechst 33342 (Sigma-Aldrich, St. Louis, USA) according to the manufacturer’s instructions. The number of TUNEL-positive cells in the layer of photoreceptors (Ph), the inner nuclear layer (INL), the ganglion cell layer (GCL) and the ciliary marginal zone (CMZ) was counted and normalized with respect to the total cell number (Hoechst stained nuclei) in Ph, INL, GCL and CMZ, respectively (Fig. S2). Each value was calculated as the mean of three sections. Each experiment was performed on at least 15 larvae per group.

### Neuroprotection study

Two nl of free neutrophic factors or conjugated neutrophic factors or saline were microinjected into the left eye of 4 dpf larvae. At 16 hpi, 2 nl of 1 M H_2_O_2_ were injected into the same eye. Larvae injected with saline at 4 dpf but non-microinjected with H_2_O_2_ were used as negative controls. Eight hours later, zebrafish larvae were fixed, paraffin-embedded, sectioned and stained with TUNEL. After staining, sections were bleached and stained for particles (Prussian blue). Only embryos positive for particles were analysed as mentioned above. Each experiment was performed on at least 15 larvae per group.

### Optokinetic response (OKR) assay

To test ocular tocixity, 2 nl of MNPs were microinjected into the left eye of 4 dpf larvae and non-injected larvae were used as controls. At 1 dpi, the OKR analysis was performed. For functional recovery analysis, 2 nl of free NTs or conjugated NTs or saline were microinjected into the left eye of 4 dpf larvae. At 16 hpi, 2 nl of 1 M H_2_O_2_ were injected into the same eye. Eight hours later, 5–9 zebrafish larvae were placed in a 3.5 mm Petri dish and embedded in 3% methylcellulose, with the dorsal side up around a small air bubble. The Petri dish was inserted inside the drum with black and white stripes rotating at ~ 6 rpm as described [28] and a video was recorded. For each larva, the number of saccades of the left eye per minute was recorded. Each experiment was performed on at least 10 larvae per group, *n* = 3.

### Data plotting and statistics

Data were plotted and analysed using GraphPad Prism. Values were reported as the mean ± standard error of the mean. Significance was set at *p* ≤ 0.05. Statistical analyses were performed by ANOVA followed by Bonferroni correction or *t* test with Welch’s correction. * is *p* < 0.05, ** is *p* < 0.01, *** is *p* < 0.001.

## Results

### Physical characterization of MNP–NT

The size distribution of MNPs was characterized by dynamic light scattering (Table 1, Fig. S3). Particles had a hydrodynamic size of 90.47 ± 2.88 nm and Polydispersity Index (PI) of 0.337 ± 0.022. MNPs exhibited a negative Z potential (− 38.72 ± 2.14 mV), due to their surface functionalization with carboxylic groups.

**Table 1:** Z potential, diameter and Polydispersion Index of naked and functionalized nanoparticles

	Z potential (mV)	Diameter (nm)	Polydispersion Index (PI)
MNP	− 38.72 ± 2.14	90.47 ± 2.88	0.337 ± 0.022
MNP–NGF	− 1.59 ± 0.21	189.57 ± 5.91	0.328 ± 0.002
MNP–BDNF	− 0.04 ± 0.03	273.07 ± 9.31	0.346 ± 0.009

Mean ± SEM. *n* = 3

For the synthesis of MNP–BDNF, we chemically linked the protein to MNPs. We found that the loading content was about 74 µg of BDNF per mg of MNPs. The hydrodynamic size of MNP–BDNF was 273.07 ± 9.31 nm and the PI was 0.346 ± 0.009. The presence of BDNF on particle surfaces was further confirmed by Z potential measurement, which shifted from − 38.72 ± 2.14 mV for naked MNPs to − 0.04 ± 0.03 mV for MNP–BDNF (the isolectric point of BDNF is 9.99).

For the synthesis of MNP–NGF, NGF was physically loaded onto MNPs by exploiting electrostatic interactions between the negatively charged MNP and the positively charged NGF (the isoelectric point of NGF 2.5S is 9.3). The loading content was about 95 µg of NGF per mg of MNPs. The hydrodynamic size of MNP–NGF was 189.57 ± 5.91 nm and the PI was 0.328 ± 0.002. The presence of NGF on particle surfaces was confirmed by Z potential measurement, which shifted from − 38.72 ± 2.14 mV for naked MNPs to − 1.59 ± 0.21 mV for MNP–NGF.

### Biological characterization of MNP–NT: biofunctionality and long-term activity of conjugated versus free NTs

**Fig. 1:**
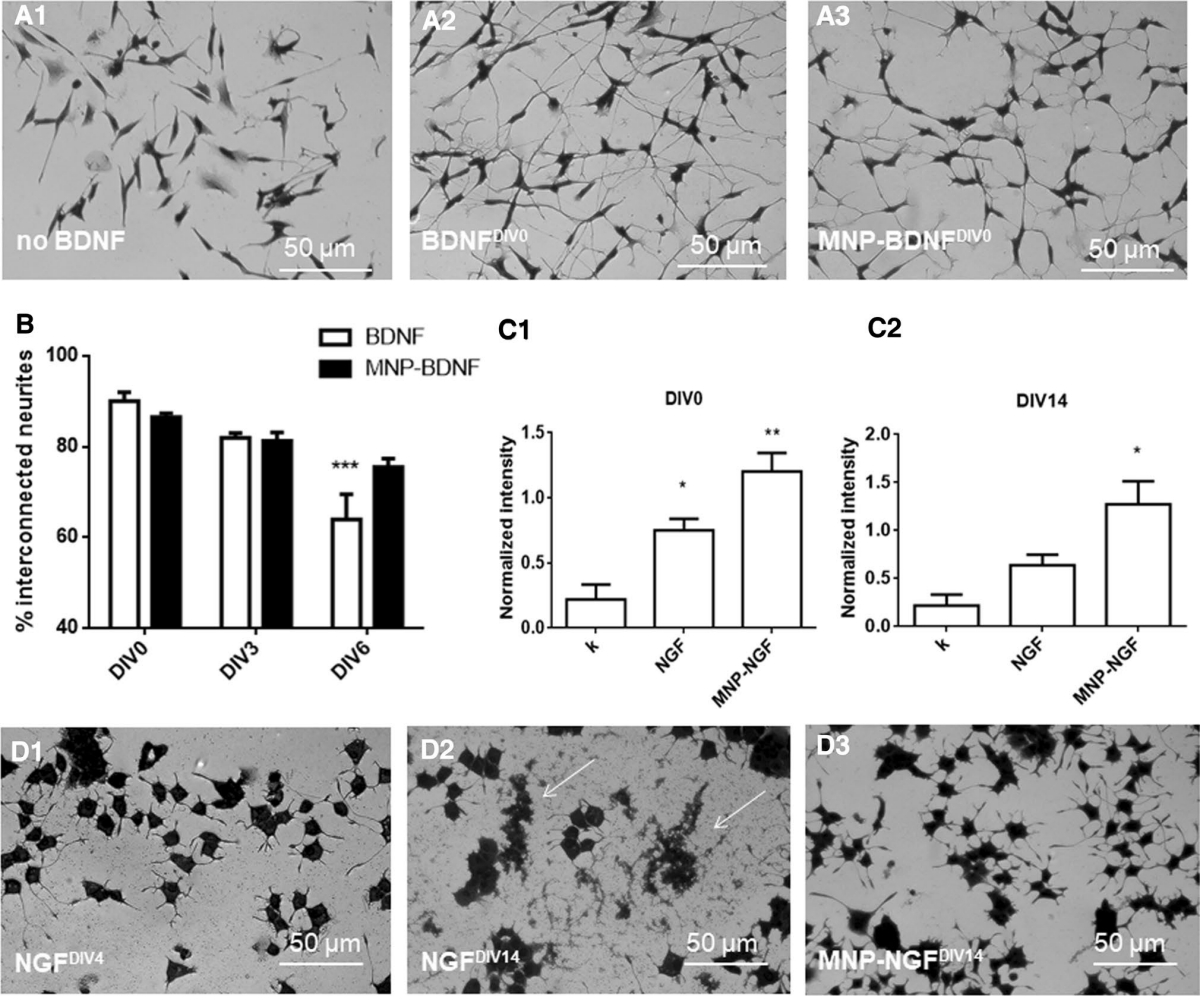
**a** RA-differentiated SH-SY5Y cells were incubated (5 days) with DMEM (*A1*) or DMEM modified with freshly frozen BDNF (100 ng/ml) (*A2*) or DMEM modified with freshly frozen MNP–BDNF (100 ng/ml) (*A3*). **b** Increase in stability of BDNF following the conjugation to MNPs. DMEM modified with BDNF or MNP–BDNF (100 ng/ml) was prepared. RA-differentiated SH-SY5Y cells were incubated (5 days) with the above-mentioned media freshly prepared (DIV0) or pre-incubated at 4 °C for 3 days (DIV3) or pre-incubated at 4 °C for 6 days (DIV6), and the percentage of interconnected neurites was calculated after fixation and staining. RA-differentiated SH-SY5Y cells were also incubated with BDNF-free DMEM as the control (not shown). *n* = 5, 2-way ANOVA followed by Bonferroni correction (all groups compared against “DIV0”), *p* < 0.0001. **c** Increase in stability of NGF following the conjugation to MNPs. Western blot analysis (p-TrkA 140 kDa and β-actin 42 kDa) carried out on extracts of PC12 cells, starved for 5 h in Optimem and treated for 5 min with the differentiation medium not modified (k), modified with NGF or MNP–NGF (100 ng/ml). Plots of p-TrkA band intensity normalized to β-actin band intensity. *C1* WB analysis performed with freshly frozen NGF and MNP–NGF samples (DIV0). *n* = 3, 1-way ANOVA followed by Bonferroni correction, *p* = 0.003. *C2* WB analysis performed with NGF and MNP–NGF samples pre-incubated for 2 weeks at 37 °C (DIV14). *n* = 3, 1-way ANOVA followed by Bonferroni correction, *p* = 0.012. **d** NGF or MNP–NGF (100 ng/ml) were incubated at 37 °C from 0 to 3 weeks. Representative images of PC12 cells treated with NGF (100 ng/ml) pre-incubated at 37 °C for 4 days (*D1*) or NGF 100 ng/ml pre-incubated at 37 °C for 14 days (*D2*) or MNP–NGF (100 ng/ml) pre-incubated at 37 °C for 14 days (*D3*). Other time points are shown in additional materials, Fig. S4

The next step was to demonstrate that the conjugation process did not alter protein biofunctionality. We validated the ability of BDNF signalling to induce the neurite network [29]. The human neuroblastoma cell line SH-SY5Y was differentiated in the presence of retinoic acid (RA). RA-differentiated cells showed an extensive neurite network when incubated with BDNF or MNP–BDNF (100 ng/ml), in contrast to BDNF-free incubation (Fig. 1a). In the BNDF-free sample, the population of interconnected neurites (i.e., neurites connected with at least one other neurite) was about half (47.15 ± 3.86%). In contrast, in the BDNF and MNP–BDNF groups, most neurites were interconnected (89.57 ± 2.45 and 87.15 ± 0.642, respectively) and these values were not statistically different (*p* > 0.05). Similarly, we validated the ability of NGF to trigger PC12 differentiation in a neuron-like phenotype. Functional analysis showed that PC12 cells incubated for 4 days in reduced medium modified with NGF or MNP–NGF (100 ng/ml) showed the same differentiated phenotype as long neurites (Fig. S4, NGF^DIV0^ and MNP–NGF^DIV0^), in agreement with our previous observations [24–26]. Molecular analysis confirmed this result. PC12 cell extracts were analysed by Western blot to detect the endogenous levels of the receptor TrkA activated by NGF (i.e., phosphorylated at tyrosine 490), confirming that both NGF and MNP–NGF induce NGF receptor phosphorylation (Fig. 1C1).

The stability of the free and conjugated neurotrophic factors was examined in different conditions. BDNF (blood half-life is 0.92 ± 0.04 min [30]), was incubated in DMEM at 4–8 °C from DIV0 (freshly frozen) to DIV6. BDNF or MNP–BDNF samples were collected at different time points and tested for their ability to induce the neurite network in RA-differentiated SH-SY5Y cells. NGF (blood half-life is 7.2 ± 0.3 min [30]) was incubated in protease-rich serum at 37 °C from 0 (freshly frozen) to DIV14. NGF or MNP–NGF samples were collected at different time points and molecular and functional analyses were performed to test their ability to induce PC12 cell differentiation.

The results demonstrated that the conjugated neurotrophic factors were significantly more stable than the free factors. Figure 1b shows the number of interconnected neurites in RA-differentiated SH-SY5Y cells incubated with free or conjugated BDNF. Statistical analyses were performed by comparing groups with freshly frozen samples (DIV0). We found a decrease in the activity of free BDNF over time, which reached statistical significance in the DIV6 sample. In contrast, no differences had been detected with conjugated BDNF by day six. Similarly, we observed that the bioactivity of free NGF quickly decreased over time, and cellular debris, which forms following cell death due to the lack of the cell differentiation stimulus mediated by NGF, was present in the cultures starting with DIV4 (Fig. 1D1). Cellular debris accumulated in the following time points (Fig. S4), until reaching complete cellular degeneration in cultures treated with NGF pre-incubated for 14 days (Fig. 1D2). In sharp contrast, in cultures treated with conjugated NGF DIV4-DIV14, PC12 cells exhibited long neurites without morphological differences from control cultures differentiated with freshly frozen NGF or freshly frozen MNP–NGF (Fig. S4). Cellular debris or signs of degeneration were absent, even at the last time point (Fig. 1D3). Molecular analysis confirmed that the 14-day pre-incubated free NGF lost its ability to induce TrkA phosphorylation (*p* > 0.05), in sharp contrast to the 14-day pre-incubated conjugated NGF, which was still able to activate the receptor (*p* < 0.05) (Fig. 1C2).

### Damage to GCL induced by oxidative stress

To induce retina damage, we IVT injected 2 nl (approximately 10% of larva vitreous volume) of H_2_O_2_ (0.25–1 M) into 5 dpf larvae. First, we performed time course experiments, to evaluate the time point corresponding to the maximum damage, following treatment with 2 nl of 1 M H_2_O_2_. We analysed the damaged retina 2, 4, 8 and 24 hpi. We found that the population of TUNEL-positive cells rapidly increased in the first hours, until reaching a peak at 8 hpi. The damage was partially restored 1 day after H_2_O_2_ injection because of the intrinsic regeneration capabilities of the developing larvae [31] (Fig. S5). Thus, all further experiments were performed by analysing the retina 8 h after damage induction.

**Fig. 2:**
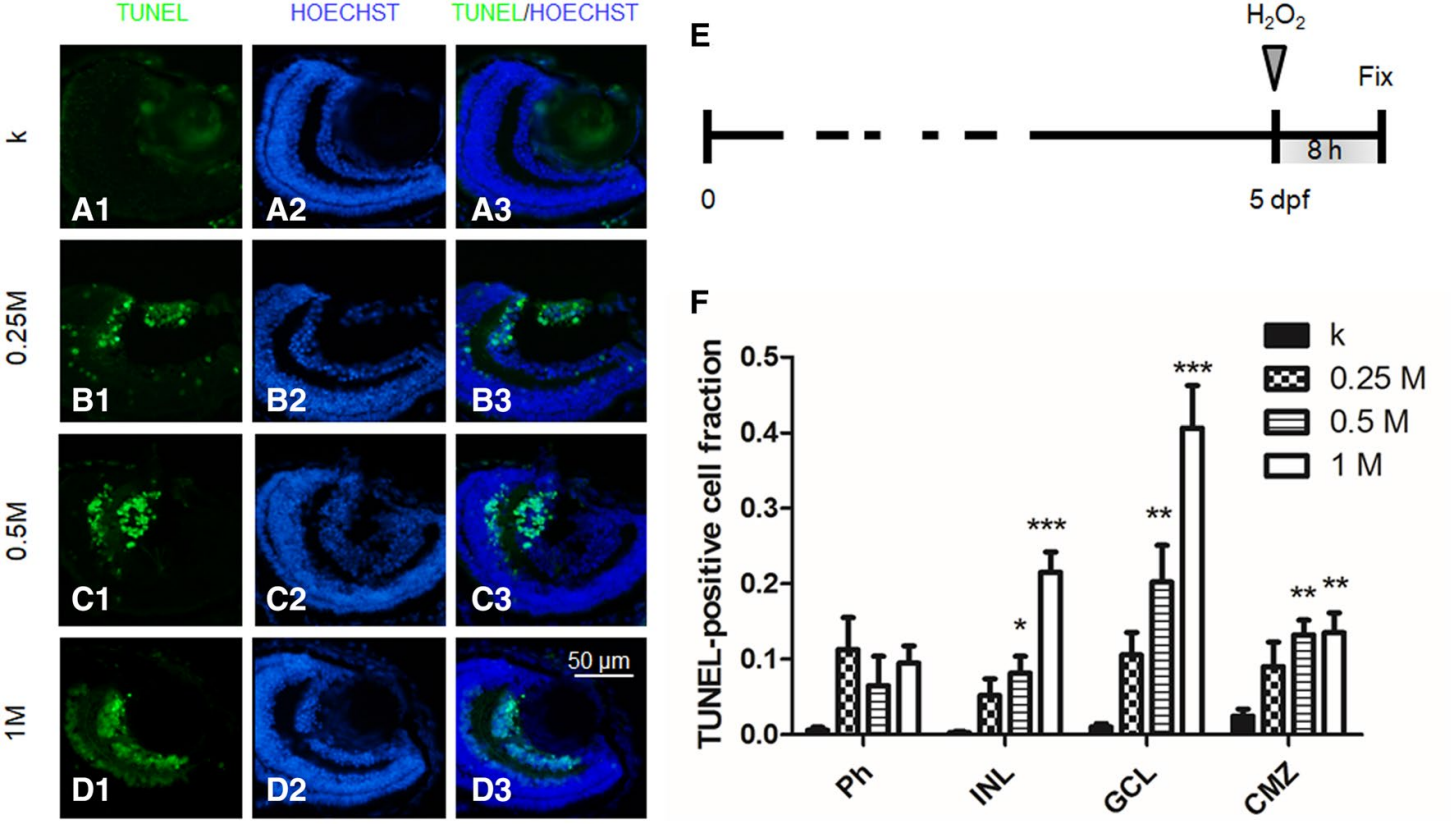
5 dpf larvae injected with 2 nl of various doses of H_2_O_2_ and fixed 8 hpi. **a**–**d** TUNEL and Hoechst staining: representative sections of larvae injected with 0 (k), 0.25 M, 0.5 M or 1 M H_2_O_2_. **e** Time course of the experiment. **f** Quantitative analysis of TUNEL-positive cells in Ph, INL, GCL and CMZ cell layers for the different doses. *n* ≥ 15 (embryos), 1-way ANOVA followed by Bonferroni correction (all groups compared against k group). Ph: *p* = 0.04. INL: *p* < 0.0001. GCL: *p* < 0.0001. CMZ: *p* = 0.0003

Next, we performed dose–response experiments. We IVT injected 2 nl of 0.25, 0.5 and 1 M H_2_O_2_ and the same volume of saline as the control experiment (k). We found a dose–response behaviour, with TUNEL-positive cells increasing in number with the hydrogen peroxide dosage. The quantitative analysis was performed by counting the fraction of TUNEL-positive cells in Ph, INL, GCL and CMZ. Results highlighted that the damage was especially localized at the level of GCL (Fig. 2). No statistically significant damage to photoreceptors, compared to the control, was observed at any concentration tested. A concentration of 0.25 M did not induce statistically significant damage in any cell layer. Conversely, concentrations of 0.5 M and 1 M induced significant damage in INL, GCL and CMZ. In particular, the 1 M concentration, which induced cell apoptosis at 40.2 ± 5.2% levels in GCL, was considered a good model to validate the neuroprotective effects of the nanoformulations developed in this study (Fig. 2).

### Localization of IVT-injected MNPs

**Fig. 3:**
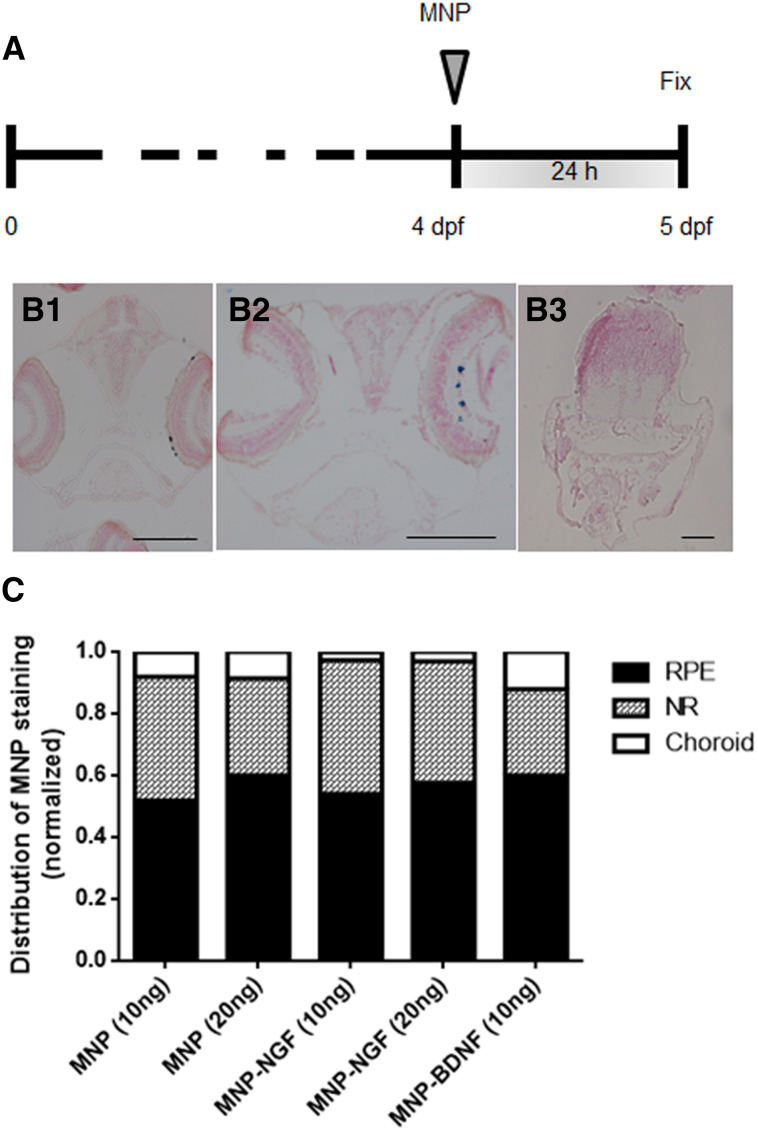
Larvae (4 dpf) were injected with the nanoparticles, and localization was studied 24 hpi (**a**). Representative images of particles localized in RPE (*B1*) or in the NR (*B2*). Particles never localise outside the ocular tissues (*B3*). Particles are stained blue (Prussian blue). The bars are 100 µm. **c** Normalized distribution of MNP staining in the NR, RPE and the choroidal layer. *n* > 15, 2-way ANOVA, *p* = 0.27

Two nl (containing 10 or 20 ng) of MNPs were IVT injected into 4 dpf larvae. Localization was studied at 24 hpi (Fig. 3a). Particles were found to localise in the retina, mainly distributed in the NR (Fig. 3B2) and RPE (Fig. 3B1), but a small fraction were also found in the choroid. Particles were never found in the controlateral eye or in other tissues (Fig. 3B3). As the surface coating is known to influence particle localization [22], we tested whether the NT conjugation altered the localization profile. Quantitative data analysis confirmed that MNP–NGF and MNP–BDNF had an identical localization profile of naked MNPs (*p* = 0.27), suggesting that NT conjugation or the dose do not change the fate of MNPs.

**Fig. 4:**
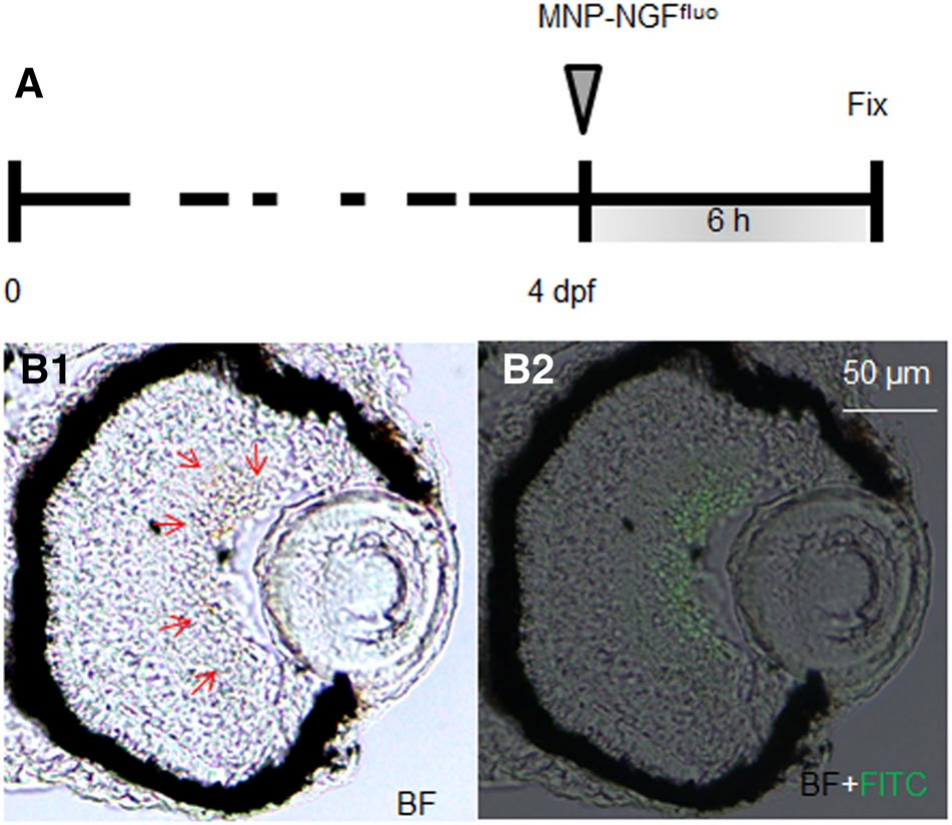
NGF was labeled with Alexa Fluor 488 (NGF^fluo^). MNP–NGF^fluo^ were injected into larvae (4 dpf) and the localization of MNPs and NGF^fluo^ was studied 6 hpi. MNPs (brown signal shown by red arrows in *B1*) and NGF^fluo^ (green staining in *B2*) were found to co-localise in the GCL

To study the localization kinetics of the conjugated MNPs, we used our previously published protocol, which consists in the fluorescent labelling of NGF with Alexa Fluor 488 by a covalent approach (NGF^fluo^) [24]. Figure 4 shows a section of 4 dpf larva injected with 2 nl of MNP–NGF^fluo^ (16 ng MNPs, 0.5 ng NGF^fluo^) at 6 hpi. MNPs (brown signal shown by red arrows, Fig. 4a) were found to co-localise with NGF^fluo^ (green fluorescence, Fig. 4b) in GCL and no fluorescence was detected in the vitreous. Our data thus suggest that MNPs efficiently carry their protein load from the vitreous chamber toward the retina.

### Conjugated NTs protect GCL from damage induced by oxidative stress

**Fig. 5:**
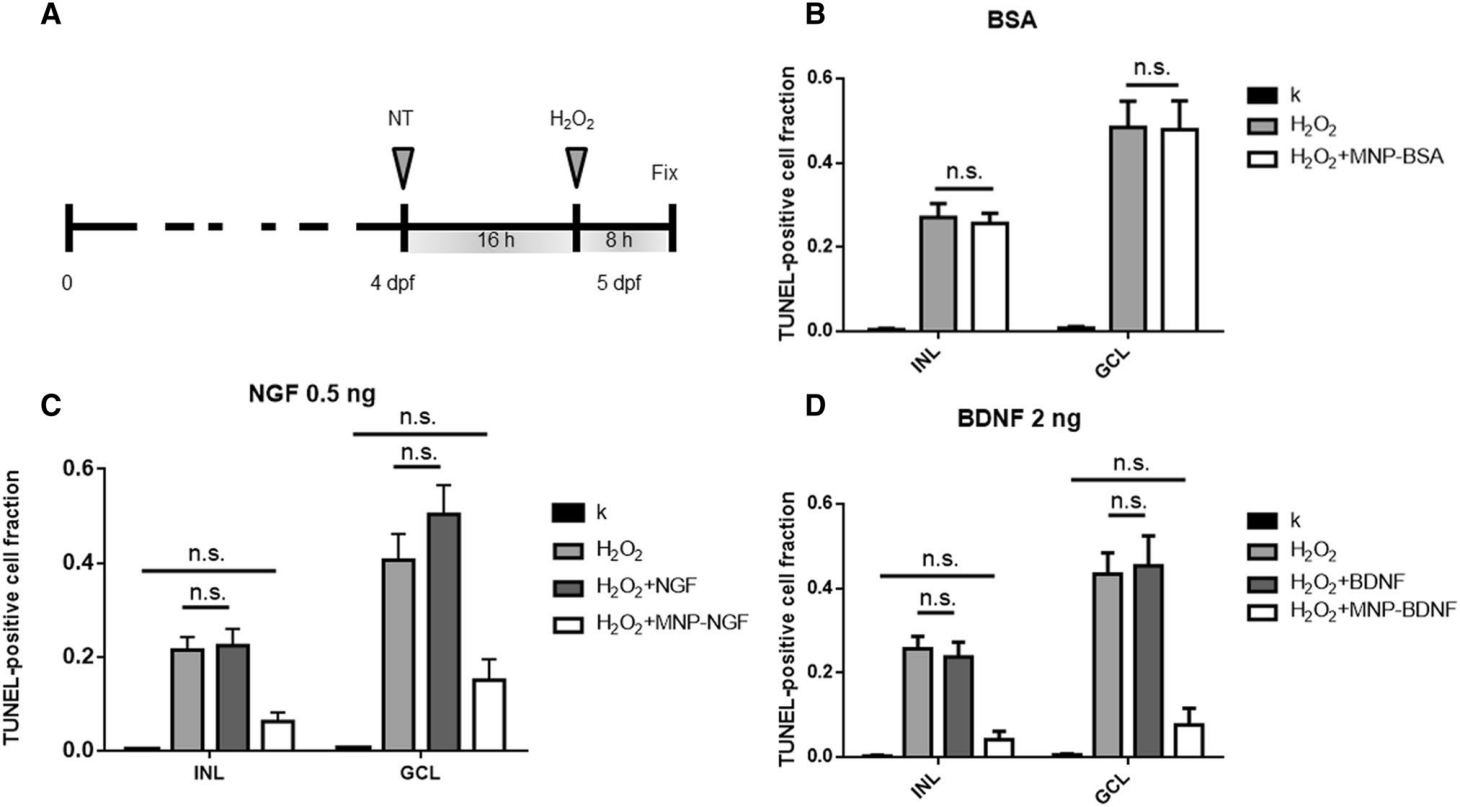
Larvae (4 dpf) were injected with the neuroprotective drug. Sixteen hours later, 2 nl of 1 M H_2_O_2_ were injected and larvae were fixed 8 h later (**a**). **b** Validation of control particles, which do not carry a neuroprotective drug (MNP-BSA). *n* ≥ 15, 1-way ANOVA followed by Bonferroni correction. INL: *p* < 0.0001. GCL: *p* < 0.0001. **c** Validation of particles, which carry 0.5 ng of NGF (MNP–NGF). *n* ≥ 15, one-way ANOVA followed by Bonferroni correction. INL: *p* < 0.0001. GCL: *p* < 0.0001. **d** Validation of particles, which carry 2 ng of BDNF (MNP–BDNF). *n* ≥ 15, 1-way ANOVA followed by Bonferroni correction. INL: *p* < 0.0001. GCL: *p* < 0.0001. **b**–**d** Show pairs that are not statistically significant (ns)

4 dpf larvae were IVT injected with free or conjugated protein and the damage was induced 16 h later by injecting H_2_O_2_ (Fig. 5a). Larvae IVT injected with saline at 4 dpf and injected (16 h later) with H_2_O_2_ or not injected, were used as the positive (H_2_O_2_) and negative (k) controls, respectively. Figure 5 shows experimental data related to GCL and INL, where most of the damage induced by H_2_O_2_ was observed (full data in Figs S6–8).

It has been reported that iron oxide MNPs may generate ROS through the Fenton and Haber–Weiss reactions [32]. In contrast, other studies have shown the capacity of MNPs to scavenge free radicals, thus attenuating oxidative damage induced by H_2_O_2_ [33]. Therefore, we decided to first ascertain whether MNPs itself interfered with oxidative stress in our model. We produced MNPs conjugated to BSA, which do not carry any neuroprotective stimulus, and injected 2 nl of dispersion into the vitreous (24 ng MNPs, 1.8 ng BSA). The average fraction of TUNEL-positive RGCs was 0.008 ± 0.004 in the saline-treated group (k). The treatment with H_2_O_2_ induced a significant increase in apoptosis, and the fraction of TUNEL-positive RGCs was 0.484 ± 0.063 (*p* < 0.0001). The pre-treatment with MNP-BSA had no effect, since the fraction of TUNEL-positive RGCs was 0.479 ± 0.069, i.e., not statistically different from the H_2_O_2_-treated group (*p* > 0.05). Similarly, the MNP–BSA pre-treatment did not influence the apoptosis level in INL: the fraction of TUNEL-positive cells was 0.27 ± 0.033 and 0.256 ± 0.024 in the H_2_O_2_-treated and MNP–BSA-pre-treated/H_2_O_2_-treated groups, respectively (not statistically different, *p* > 0.05), but were statistically different compared to k group (0.004 ± 0.003, *p* < 0.0001). We concluded that particles per se had a negligible effect on the apoptosis level.

Next, we tested the neuroprotection of the nanoformulations developed here, using 0.5 ng of free and conjugated NGF (Fig. 5c). We found that the IVT injection of free NGF did not prevent the damage induced by H_2_O_2_ in GCL; the fraction of apoptotic cells was 0.504 ± 0.062 and 0.406 ± 0.056 for the NGF-pre-treated/H_2_O_2_-treated and the H_2_O_2_-treated groups, respectively (*p* > 0.05). In sharp contrast, the conjugated NGF totally prevented the H_2_O_2_-induced damage because the apoptosis level in the MNP–NGF-pre-treated/H_2_O_2_-treated group (0.151 ± 0.044) was not statistically different from the control k group (0.005 ± 0.003) (*p* > 0.05). We also counted the number of RGCs and we found a decrease in the fraction of RGCs after the H_2_O_2_-induced damage (0.78 ± 0.05, *n* > 15). The pre-treatment with the free protein reduced the RGC loss, however, the number of RGCs after damage induction was not statistically different from the H_2_O_2_-treated group (0.911 ± 0.049, *n* > 15, *p* > 0.05). In line with previous data, the pre-treatment with the conjugated NGF totally prevented the RGC loss due to the oxidative stress (1.04 ± 0.053%, *n* > 15, *p* < 0.001).

A similar result was achieved in INL, where the apoptosis level caused by H_2_O_2_ (0.215 ± 0.027) was not statistically different from the level found in the NGF-pre-treated/H_2_O_2_-treated group (0.224 ± 0.035) (*p* > 0.05). On the other hand, the average number of TUNEL-positive cells in the MNP–NGF-pre-treated/H_2_O_2_-treated group (0.063 ± 0.019) was similar to the k group (0.003 ± 0.002) (*p* > 0.05).

In conclusion, we found clear evidence that the free NGF does not protect GCL and INL from the damage induced by H_2_O_2_, in sharp contrast to the conjugated NGF. This is not surprising because the NGF dose used here was about 10% of the dose (~ 0.4 μg NGF/μL of vitreous volume) usually required for effective neuroprotection of the retina from degeneration [34, 35]. Indeed, the conjugation of NGF to MNPs led to a drastic reduction in the dose required for neuroprotection.

Excellent results were also achieved by testing 2 ng of recombinant BDNF (Fig. 5d). RGC protection from damage was achieved by the MNP–BDNF pre-treatment, with similar levels of TUNEL-positive cells to the control k (0.076 ± 0.039 and 0.005 ± 0.003 for BDNF-pre-treated/H_2_O_2_-treated and k groups, respectively, *p* > 0.05), while the free protein had no effect (0.434 ± 0.051 and 0.453 ± 0.071 for H_2_O_2_-treated and BDNF-pre-treated/H_2_O_2_-treated groups, respectively, *p* > 0.05). Similarly, no significant apoptosis level was detected in INL in the group receiving the MNP–BDNF pre-treatment (0.042 ± 0.012 and 0.003 ± 0.002 for BDNF-pre-treated/H_2_O_2_-treated and k groups, respectively, *p* > 0.05), while no neuroprotection was actuated by the free protein (0.257 ± 0.03 and 0.237 ± 0.035 for BDNF-pre-treated/H_2_O_2_-treated and H_2_O_2_-treated groups, respectively, *p* > 0.05).

**Fig. 6:**
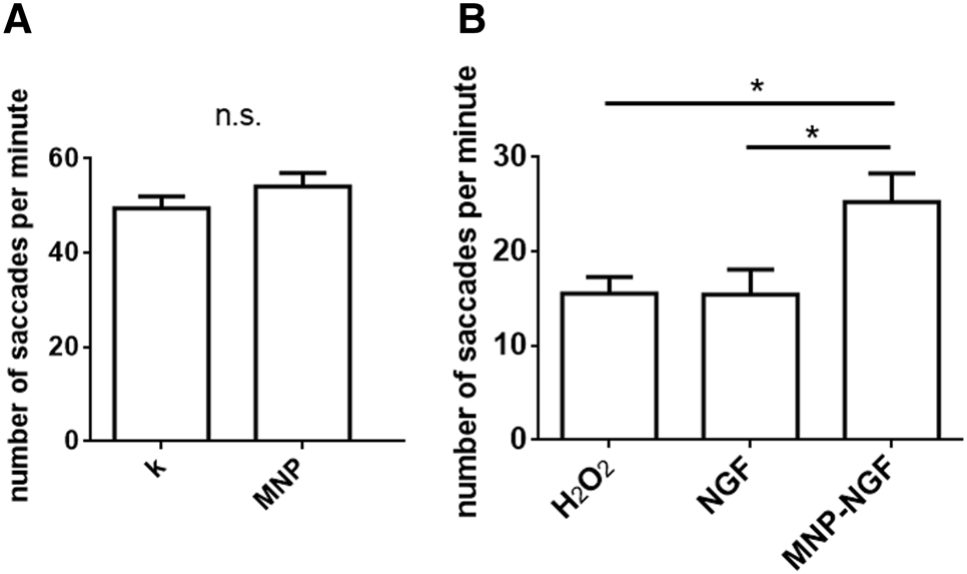
Optokinetic response assay. **a** Larvae were treated as shown in Fig. 3a. The injection of MNPs (20 ng) did not induce any change in OKR. *n* ≥ 35. *T* test, *p* = 0.22. **b** Larvae were treated as shown in Fig. 5a. The conjugated NGF but not the free NGF decreased visual function loss. *n* ≥ 30, one-way ANOVA followed by Bonferroni correction, *p* = 0.0095

The visual behaviour of larvae was studied by examining the optokinetic response (OKR), i.e. larval eye movements (saccades) in response to rotating illuminated stripes, which can be accurately measured in developing larvae by 4 dpf [28]. The number of saccades per minute in non-injected larvae was 49.41 ± 2.57, which was not statistically different from the value of 54.12 ± 2.83 recorded in larvae IVT injected with 20 ng of MNPs (*n* > 35, *p* = 0.22), thus confirming the absence of optical toxicity arising from the MNPs (Fig. 6a). The injection of H_2_O_2_ led to a loss of visual function (15.53 ± 1.70 saccades/min). No difference was found compared to the NGF-pre-treated/H_2_O_2_–treated groups (15.44 ± 2.64 saccades/min). However, a moderate protection against visual loss was recorded for the MNP–NGF-pre-treated/H_2_O_2_-treated groups (25.24 ± 3.04), which reached statistical significance compared to the H_2_O_2_-treated group (*p* < 0.05) and to the NGF-pre-treated/H_2_O_2_-treated groups (*p* < 0.05) (Fig. 6b).

## Discussion

This study tested the neuroprotective effect of NTs conjugated to MNPs versus the free factors. For the synthesis of MNP–BDNF, we covalently linked the protein to MNPs, according to a protocol we had already optimized for other growth factors such as GDNF [26] and the vascular endothelial growth factor (VEGF) [22].

Compared to naked MNPs, the hydrodynamic size of MNP–BDNF tripled (Table 1). This increase probably depends on cross-linking events among two or a few particles rather than particle aggregation or precipitation (the PI of MNP–BDNF and naked MNPs are similar, Table 1). This is a common mechanism already observed by performing the covalent synthesis of other proteins, such as BSA, GDNF [26] and VEGF [22]. In fact, proteins have carboxylic groups that can be activated by EDC chemistry, leading to a degree of particle cross-linking, which is usually higher for covalent than for non-covalent chemistry [25]. For the synthesis of MNP–NGF, we used a non-covalent approach. In fact, in previous studies, we compared the physical load of NGF versus the chemical link of NGF to MNPs. Both strategies were very efficient for protein binding, leading to very stable conjugates, even after the addition of the cell growth medium [24], however, the chemical link impaired NGF bioactivity [26]. According to our evidence, physical binding should be the elective choice for NGF conjugation. Interestingly, this behaviour is protein-specific and cannot be generalized. For example, we observed that the chemical link of the GDNF to the MNPs totally preserved the protein bioactivity, in sharp contrast to NGF [26]. Compared to naked MNPs, the hydrodynamic size of MNP–NGF doubled (Table 1). Similarly to MNP–BDNF, the cross-linking events among a few particles are probably responsible for such an increase (the PI of MNP–NGF decreased compared to that of naked MNPs, Table 1). The presence of proteins on particle surfaces was confirmed by absorbance, Z potential (Table 1) and protein bioactivity measurements (Fig. 1).

In this study, we performed comparative biological studies on zebrafish larvae, whose retina were damaged by the oxidative stress induced by IVT injection of hydrogen peroxide. The reactive oxygen species (ROS) take part in the pathogenesis of anterior and posterior eye segment diseases in adults [36]. Superoxide is generated directly from a reduction in oxygen and then converted into hydrogen peroxide. H_2_O_2_ can readily penetrate cell membranes and generate the most reactive form of oxygen, the hydroxyl radical. Low levels of ROS production are required for the physiological control functions of the cells [37]. However, increased ROS production, oxidative retina damage and an imbalance between pro-oxidant and antioxidant capacities are believed to be crucial factors for glaucoma onset [38].

We used zebrafish embryos as a model system. Zebrafish have become a popular vertebrate model to study a variety of human diseases and are more recently becoming a valuable tool for the study of human ophthalmological disorders [39]. The visual system of the zebrafish is fundamentally similar to that of human subjects [40], and the layer of the Ph, INL, and GCL share the same structure as the human eye (Fig. S2). In contrast to humans, larval fish, frogs and birds have CMZ, which is located at the extreme periphery of the maturing neural retina and consists of retinal stem and progenitor cells [41]. The retinal structure of fish starts to develop from 32 hpf (hours post fertilization) and is complete within 5 dpf [42].

In this study, the damage was induced by intravitreally injecting 2 nl of 1 M H_2_O_2_ in 5 dpf larvae. One of the disadvantages of using zebrafish to investigate ocular diseases affecting cell survival is the notable capacity for retinal cell regeneration, including the GCL [43]. We performed timecourse experiments to investigate the optimal time point for damage evaluation from the time of damage induction. We found that the apoptosis level quickly increased in the initial hours, reaching a peak at 8 hpi, followed by a reduction over time due to the intrinsic regeneration capability of the larvae (Fig. S5). After 8 hpi, we detected high levels of apoptosis in GCL (40.2 ± 5.61%), moderate levels in INL (21.5 ± 2.73%), low levels in CMZ (13.5 ± 2.63%) and negligible effects on the Ph layer (Fig. 2f). We then tested the potential of the nanoformulations developed in this study to prevent RGC damage induced by oxygen peroxide. Given the involvement of oxidative stress in the pathophysiology of glaucoma, neuroprotective or antioxidant therapies have been designed to prevent RGC loss and have been already tested pre-clinically [44]. Neuroprotective drugs should increase cellular resistance to the deleterious effects of oxidative stress, while antioxidant drugs target the oxidative stress itself.

Ongoing studies are investigating the beneficial effects of antioxidants on glaucoma, such as α-tocopherol and Gingko (NCT01544192), or vitamin D in combination with memantine, an NMDA-receptor antagonist (NCT01409694), however, the therapeutic efficacy appears to be limited [44]. Despite the success of preclinical studies, clinical trials rarely test the neuroprotective effects of neurotrophic factors because of the difficulties with drug delivery. Here, we validated the neuroprotective effect of conjugated NTs, in comparison to free factors. Histological observations revealed that 0.5 ng of NGF 2.5S and 2 ng of recombinant BDNF conjugated to iron oxide MNPs induced total neuroprotection against RGC loss. The same amount of free factors had no effect on reducing the apoptosis level induced by ROS generation (Fig. 5). The conjugated NGF, but not the free protein, also attenuated the impairment of visual function caused by the IVT injection of H_2_O_2_ (Fig. 6b).

We demonstrated that the conjugated MNPs actuated their neuroprotective action by combining two effects. First, the conjugation of the neurotrophic factors to MNPs increases their stability, preserves and thus improves their activity. Our data suggest that NTs conjugated to MNPs degrade more slowly than free NTs, and MNPs extend the half-life of NTs (Fig. 1). This result is consistent with data reported in the literature. Ziv-Polat and colleagues showed that NGF, GDNF or FGF-2 conjugated to iron oxide nanoparticles were significantly more stable in cell cultures and in media than the free factors. This thus suggests that the coupling of proteins to nanoparticles prolongs their half-life and enhances their activity in vitro and in vivo, by protecting them against proteolytic enzymes and inhibitors in the serum or secreted by the cells [45]. Similarly, Marcus and colleagues showed that NGF conjugated to iron oxide MNPs undergoes slower degradation than free NGF, and conjugation improved the NGF function in inducing neuronal differentiation [46]. Here, we confirm that the conjugation of NTs to MNPs strongly prolongs NT biological activity over time.

Second, MNPs carry the neurotrophic factors in the retina, thus preventing their rapid mass loss due to physiological elimination. In a previous study, we demonstrated that iron oxide MNPs are able to self-accumulate in the retina [21]. Our previous results indicated that the MNPs progressively migrate from the vitreous chamber toward the retina, and that the migration process is completed within 24 h. Here, we demonstrated that MNPs transport their cargo (NTs) to GCL at 6 hpi (Fig. 4), progressively migrating toward the NR and the RPE, where they preferentially localise at 24 hpi (Fig. 3). In fact, MNPs can be used to maintain factors in situ and to prevent the loss of exogenously administered NTs over time due to physiological elimination, which naturally occurs in the vitreous.

Our studies have shown that the use of conjugated NTs could overcome the current limitations in terms of their ocular delivery, suggesting a therapeutically effective strategy for translating pre-clinically proven benefits into clinical applications. However, the anatomical and physiological differences between the zebrafish and human eye require further experimentation on mammalian models to extend the current results to other species, and to translate them into the clinical setting.

### Electronic supplementary material

Below is the link to the electronic supplementary material.

Supplementary material 1 (DOCX 688 kb)

## Abbreviations

BSA: Bovine serum albumin
BDNF: Brain-derived neurotrophic factor
CNS: Central nervous system
CMZ: Ciliary marginal zone
DIV: Days in vitro
dpf: Days post fertilization
DMEM: Dulbecco’s modified Eagle’s medium
FGF: Fibroblast growth factor
NGF^fluo^: Fluorescently labelled NGF
FBS: Foetal bovine serum
GCL: Ganglion cell layer
GDNF: Glial cell-derived neurotrophic factor
hpf: Hours post fertilization
hpi: Hours post injection
INL: Inner nuclear layer
IVT: Intravitreal
MNP: Magnetic nanoparticle
NP: Nanoparticle
NGF: Nerve growth factor
NR: Neural retina
NT: Neurotrophin
PNS: Peripheral nervous system
Ph: Photoreceptor
PI: Polydispersion index
PLL: Poly-l-lysine
RGC: Retinal ganglion cell
ROS: Reactive oxygen species
RPE: Retinal pigment epithelium
RA: Retinoic acid
VEGF: Vascular endothelial growth factor
