# Supplementary material for: Neurotrophin-conjugated nanoparticles prevent retina damage induced by oxidative stress
Source: Cell Mol Life Sci. 2017 Nov 2;75(7):1255–67. doi: 10.1007/s00018-017-2691-x (PMC5843686; doi:10.1007/s00018-017-2691-x)
Supplement: Supplementary file 1 — Supplementary material 1 (DOCX 688 kb) [file 18_2017_2691_MOESM1_ESM.docx]

Supporting Information


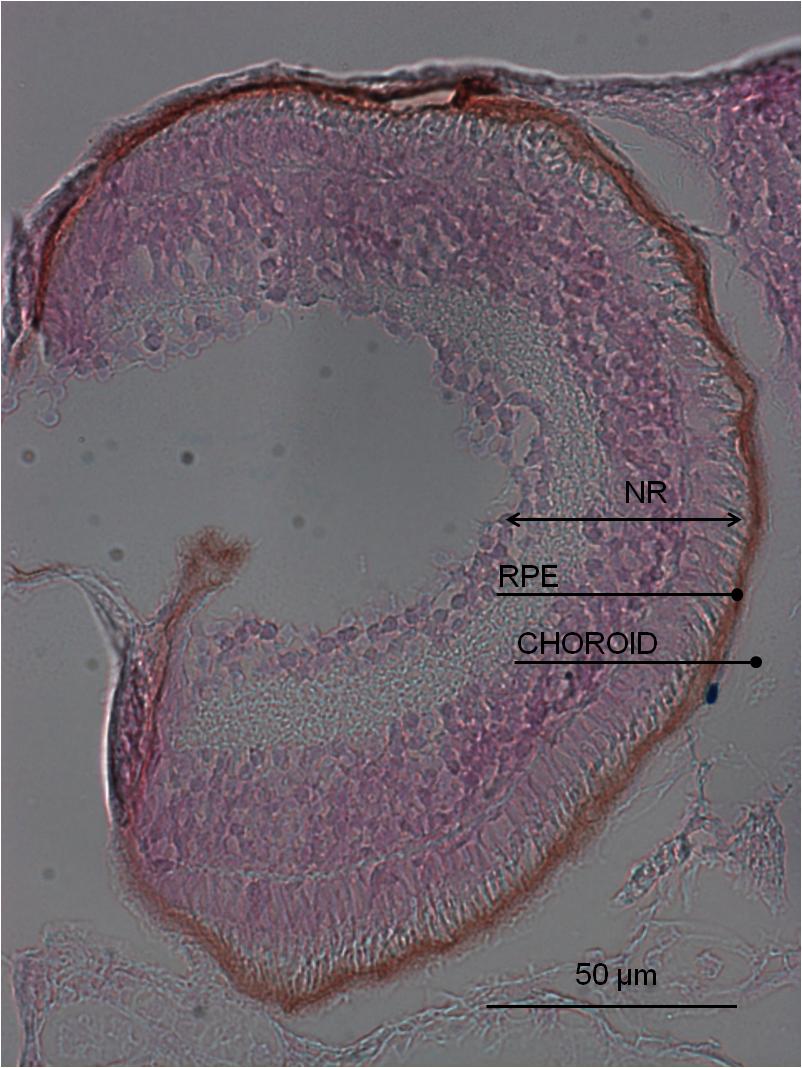


Figure S1: Retina, RPE and choroid are shown in an eye section of 5 dpf larva.


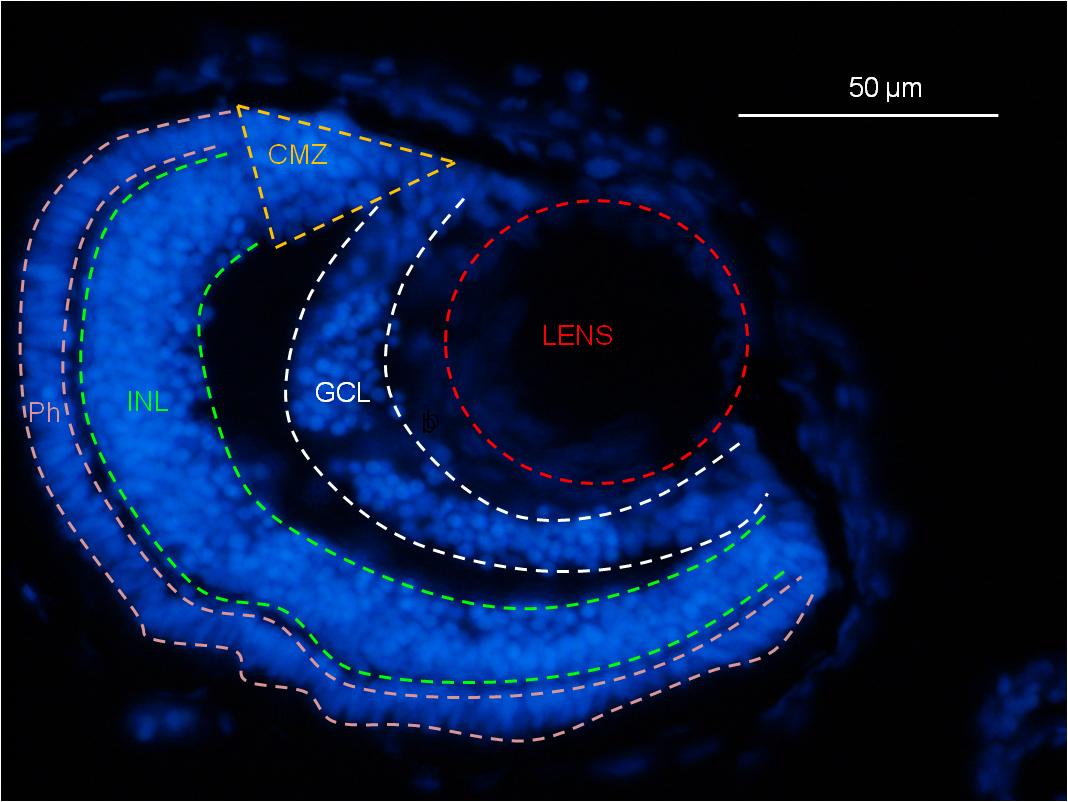


Figure S2: The boundaries of lens (red dotted lined), GCL (white dotted lines), INL (green dotted lines), Ph (pink dotted lines) and CMZ (yellow dotted lines) are schematically represented in an eye section of 5 dpf larva.


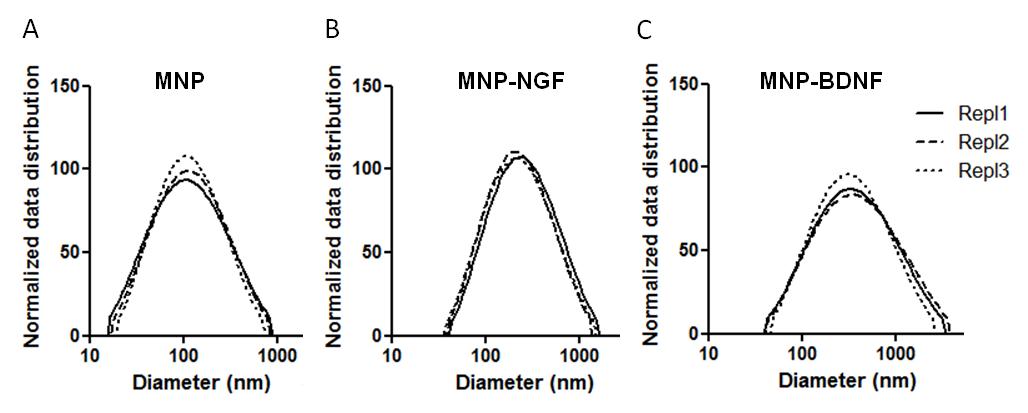


Figure S3. DLS analysis of MNP (A), MNP-NGF (B) and MNP-BDNF (C). n=3


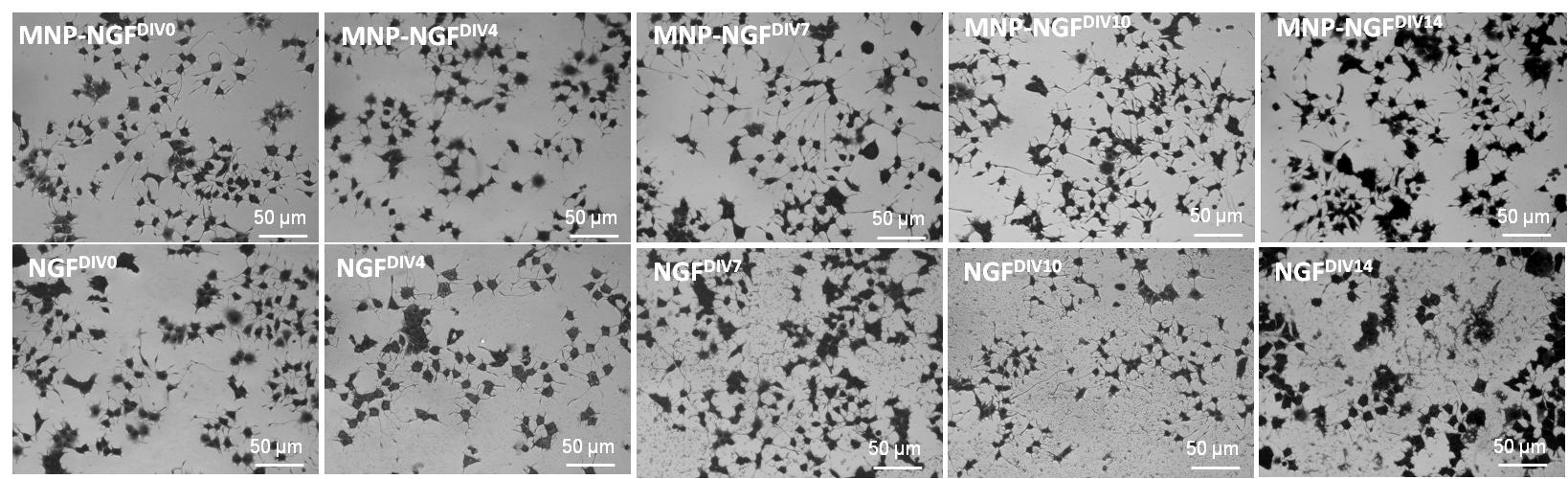


Figure S4. PC12 differentiation was induced by using NGF or MNP-NGF 100 ng/ml pre-incubated at 37°C from 0 to 2 weeks. Images show that the pre-incubation of NGF at 37°C in protease rich medium induce a fast degradation of the protein: cellular debris appears in the DIV4 sample, followed by progressive cellular degeneration in the following time points. In sharp contrast, all MNP-NGF samples pre-incubated at 37°C in protease rich medium (top) look similar to the control and cellular debris or degeneration are absent, even in the DIV14 sample.


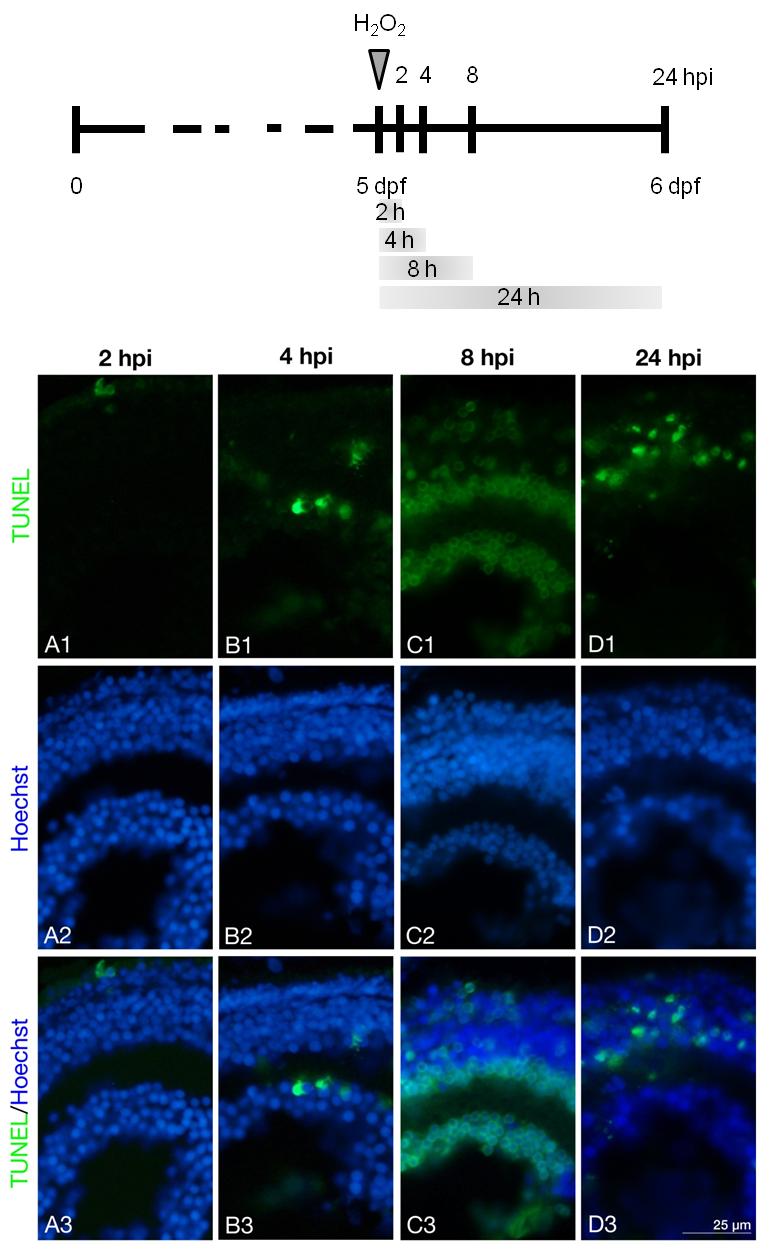


Figure S5. Representative images of 5 dpf larvae injected with 2 nl of 1 M H_2_O_2_ and fixed 2 or 4 or 8 or 24 hours post injection (A, B, C and D, respectively): TUNEL staining (1) and Hoechst staining (2) and their merge (3).

Figure S6. Larvae (4 dpf) were injected with the neuroprotective drug. Sixteen hours later, 2 nl of 1M H_2_O_2_ were injected and larvae were fixed 8 hours later. Validation of control particles which do not carry a neuroprotective drug (MNP-BSA). n>=15. 1-way ANOVA followed by Bonferroni correction. Ph: p=0.006. INL: p<0.0001. GCL: p<0.0001. CMZ: p=0.355. n.s.: not significant.

 Figure S7. Larvae (4 dpf) were injected with the neuroprotective drug. Sixteen hours later, 2 nl of 1M H_2_O_2_ were injected and larvae were fixed 8 hours later (A). Validation of particles, which carry 0.5 ng of NGF (MNP-NGF). n>=15. 1-way ANOVA followed by Bonferroni correction. Ph: p=0.001. INL: p<0.0001. GCL: p<0.0001. CMZ: p<0.0001. n.s.: not significant.

Figure S8. Larvae (4 dpf) were injected with the neuroprotective drug. Sixteen hours later, 2 nl of 1M H_2_O_2_ were injected and larvae were fixed 8 hours later. Validation of particles which carry 2 ng of BDNF (MNP-BDNF). n>=15. 1-way ANOVA followed by Bonferroni correction. Ph: p=0.069. INL: p<0.0001. GCL: p<0.0001. CMZ: p<0.0004. n.s.: not significant.
